# Review and Updates on the Treatment of Refractory and Super Refractory Status Epilepticus

**DOI:** 10.3390/jcm10143028

**Published:** 2021-07-07

**Authors:** Yazeed S. Alolayan, Kelly McKinley, Ritwik Bhatia, Ayham Alkhachroum

**Affiliations:** 1 Department of Neurology, University of Miami Miller School of Medicine, Miami, FL 33136, USA; dr.yazeed.ola@gmail.com (Y.S.A.); ritwik.bhatia@jhsmiami.org (R.B.); 2 Neuroscience Intensive Care Unit, Jackson Memorial Hospital, Miami, FL 33136, USA; kelly.mckinley@jhsmiami.org

**Keywords:** refractory status epilepticus, super-refractory status epilepticus, treatment, electroencephalogram

## Abstract

Refractory and super-refractory status epilepticus (RSE and SRSE) are life-threatening conditions requiring prompt initiation of appropriate treatment to avoid permanent neurological damage and reduce morbidity and mortality. RSE is defined as status epilepticus that persists despite administering at least two appropriately dosed parenteral medications, including a benzodiazepine. SRSE is status epilepticus that persists at least 24 h after adding at least one appropriately dosed continuous anesthetic (i.e., midazolam, propofol, pentobarbital, and ketamine). Other therapeutic interventions include immunotherapy, neuromodulation, ketogenic diet, or even surgical intervention in certain cases. Continuous electroencephalogram is an essential monitoring tool for diagnosis and treatment. In this review, we focus on the diagnosis and treatment of RSE and SRSE.

## 1. Introduction

Status epilepticus (SE) is a critical condition that requires prompt treatment to minimize long-term consequences from continuous seizure activity. In 2015, the International League Against Epilepsy defined status epilepticus as “a condition resulting either from the failure of the mechanisms responsible for seizure termination or from the initiation of mechanisms which lead to abnormally prolonged seizures (after time point t1)… and can have long-term consequences (after time t2), including neuronal death, neuronal injury, and alteration of neuronal networks” [1]. Commonly used timepoints for t1 and t2 are five and thirty minutes in generalized seizures, respectively. If a delay in SE treatment persists beyond five minutes, or if SE is undertreated, progression to refractory status epilepticus (RSE) and super-refractory status epilepticus (SRSE) may occur [1]. RSE is defined as SE that persists despite at least two appropriately dosed parenteral anti-seizure medications (ASMs), while SRSE is SE that persists at least 24 h after the onset of continuous anesthetic medications (i.e., midazolam, propofol, pentobarbital, and ketamine), or during the weaning of these medications [2]. Delayed treatment can lead to neuronal injury, irreversible damage to vulnerable regions in the hippocampus, thalamus, and neocortex, and in some cases brain tissue hypoxia, and increased intracranial pressure [3].

The etiology of SE can be categorized into symptomatic and cryptogenic, with additional subcategories based on time course (acute, remote, and progressive) [1]. Common causes include low ASM levels, cerebrovascular disease, traumatic brain injury, intracranial tumor, central nervous system infection, autoimmune encephalitis, and toxic and metabolic derangements (i.e., alcohol withdrawal/intoxication, or hypoxia) [4]. On the cellular level, overexpression of excitatory receptors (i.e., AMPA, NMDA, glutamate, voltage-gated sodium, and calcium channels), and either downregulation, internalization, or in combination, of inhibitory receptors (i.e., GABA_A_ receptors) are thought to be reasonable explanations for benzodiazepine resistance in SE [5].

SE progresses to RSE in 12 to 48% of cases, of which 15–22% may progress to SRSE [6,7]. Some risk factors for progression to RSE include focal motor seizures at onset, nonconvulsive SE, high peak glucose level, and autoimmune encephalitis [8].Mortality in published literature ranges from 10–40% for RSE and 35–65% for SRSE [9,10,11,12,13].

## 2. Diagnostic Workup

**Figure 1:**
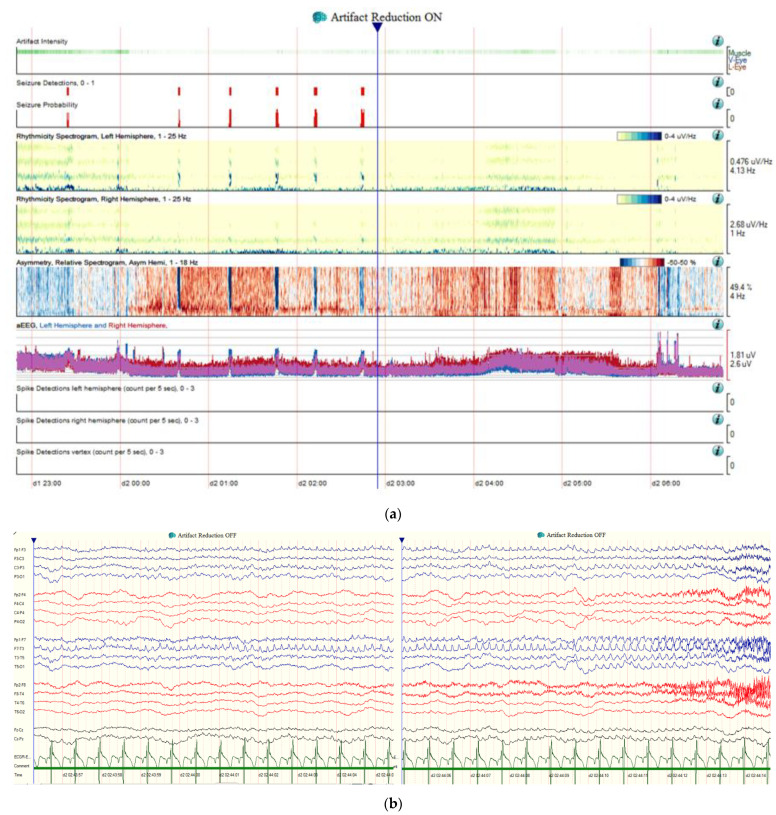
Seizure example of quantitative EEG (qEEG) and raw EEG. (**a**) Seizures on qEEG in an 81-year-old patient with history of epilepsy who presented with SE in the settings of bacteremia. The seizure detection and seizure probability are shown in an 8-h window of EEG monitoring; indicating 6 seizures. Additionally, increased rhythmicity is more pronounced on the left hemisphere compared to the right hemisphere, clear asymmetry (relative spectrogram) and increased amplitude on the left hemisphere (blue color) on an EEG during the seizures. qEEG can be used as a tool to assist clinicians with the diagnosis and the evaluation of treatment response in patients with SE. (**b**) One of the seizure events is demonstrated on the raw EEG, with left hemispheric distribution.

When evaluating a patient presenting with SE, clinicians should focus on maintaining the patient’s safety and stability (airway, breathing, and circulation). Glucose level, computed tomography (CT) scan of the head, an electroencephalogram (EEG), and basic laboratory workup including complete blood count, complete metabolic panel, and calcium level, should be obtained as soon as possible. After the acute phase of diagnosis and treatment, further workup including but not limited to brain magnetic resonance imaging (MRI), lumbar puncture, toxicology panel, serum ethanol level, and serum ASM levels should be considered [2]. It is important to note that SE can be secondary to autoimmune or paraneoplastic processes, which may require further workup such as malignancy imaging (CT or MRI scan of the chest abdomen and pelvis, ultrasound of the testicles or ovaries, positron emission tomography) and serum and cerebrospinal fluid (CSF) paraneoplastic antibody panels [14]. Continuous EEG plays a critical role for the diagnosis and treatment of patients with SE. Quantitative EEG is another tool that compresses raw EEG data using mathematical and analytical algorithms, transforming the data into graphical displays that might be easier for bedside providers to interpret and subsequently make treatment decisions [15,16] (Figure 1).

## 3. Treatment of RSE and SRSE

The definitions of RSE and SRSE incorporate key elements to consider regarding the timing and selection of ASMs and other treatment modalities. When seizures continue despite initial treatment with a benzodiazepine and one to two additional ASMs, clinicians should consider continuous anesthetics for the treatment of RSE. Clear treatment goals incorporating the risks and benefits of continuous anesthetic therapy should be defined early on using an interdisciplinary approach. It is particularly essential to bridge the gap between electrophysiologist and bedside clinician when treating patients with SE [17].

The initial goal of therapy is aimed towards eliminating or reducing seizure activity on continuous EEG. The current guidelines recommend cessation of electrographic seizures or burst suppression [2]. There are no data to support superiority of electrographic seizure control or burst suppression. However, treatment to EEG suppression was associated with lower occurrence of breakthrough seizures [18].Burst suppression is defined as an EEG pattern consisting of periods of very low brain voltage electrical activity “suppression”, and alternating periods of higher amplitude activity “burst” [19]. The literature regarding therapeutic coma of “seizure free” duration is ambiguous and not well defined. Typically, 24–48 h of seizure free activity followed by the gradual weaning of anesthetics is recommended [2,20]. More recently, Muhlhofer and colleagues published a retrospective observational cohort study involving patients with RSE that underwent therapeutic coma. The authors found that deeper (maximal anesthetic dose) and shorter duration (less than 35 h) of sedation was effective, safe, and was associated with shorter duration of mechanical ventilation and ICU length of stay [21]. More research is needed on the ideal treatment target in SE.

Clinicians should be aware of the complications of RSE and SRSE treatment. This includes hemodynamic instability, propofol infusion syndrome (bradycardia, metabolic acidosis, cardiovascular collapse, rhabdomyolysis, renal failure, and hepatomegaly in the settings of high and prolonged use of propofol), ileus, acidosis, bowel perforation (with the use of high anesthetics dosages), and increased risk of infection (including pneumonia and sepsis) [22]. It is important to monitor the patient closely with frequent vital signs and laboratory values (i.e., renal function test, liver function test, complete blood count, blood gas, bicarbonate, and ASM levels).

### 3.1. Commonly Used ASM in the Treatment of RSE and SRSE

Due to side effects and complications associated with continuous anesthetic medications, nonsedating ASMs are often initiated in tandem during the management of RSE and SRSE. Data are limited regarding general approach and drug of choice [2]. First line ASMs for the treatment of benzodiazepine resistant SE were compared in the large randomized, controlled, comparative-effectiveness Established Status Epilepticus Treatment Trial (ESETT). This trial demonstrated no difference in rates of seizure cessation (50%) among patients treated with fosphenytoin (20 mg/kg phenytoin equivalents max 1500 mg), levetiracetam (60 mg/kg max 4500 mg), or valproic acid (40 mg/kg max 300 mg). The authors reported no significant differences in side effects including hypotension, arrhythmias, or respiratory depression requiring endotracheal intubation, with only a trend toward higher rates of hypotension and endotracheal intubation with fosphenytoin compared to levetiracetam and valproic acid [23]. Other drugs studied primarily in case series have included topiramate, perampanel, brivaracetam, lacosamide, and phenobarbital, the latter of which was administered orally and facilitated successful weaning of pentobarbital [24,25,26,27,28]. Sodium channel blockers have been used for the management of KCNQ2, SCN2A, and SCN8A [29]. It is important to note that most studies addressing the treatment of SE are observational in nature, and are not randomized clinical trials, mainly targeting convulsive SE [23,30]. Please refer to Table 1 for the most commonly used ASM in RSE and SRSE.

### 3.2. Commonly Used Anesthetics in the Treatment of RSE and SRSE

Although there are no high-quality data for the optimal anesthetics for the treatment of RSE and SRSE, benzodiazepines, propofol, barbiturates, and ketamine have been among the most commonly used. Traditionally, both midazolam and propofol have been used for decades as first-line continuous anesthetics for RSE and SRSE [18]. When one agent is initiated and up titrated without achieving therapeutic goals, the addition of another agent should be considered [17,31]. Additionally, clinicians should consider inhaled anesthetics such as isoflurane and desflurane in prolonged cases of SRSE [32]. However, hippocampal atrophy was reported with the use of inhaled anesthetics [33]. In this section, we discuss the most commonly used anesthetics for the treatment of RSE and SRSE (Table 2).

#### 3.2.1. Midazolam

Midazolam (Class IIa, level B) is a fast acting 1,4-benzodiazepine, that exerts its therapeutic effect by binding to and enhancing the activity of the GABA_A_ receptor, with a dose-dependent effect of lowering seizure recurrence and mortality at hospital discharge [18,34]. Advantages of midazolam include its fast onset of action and relatively short half-life allowing for a faster off titration than other anesthetics such as pentobarbital. Additionally, midazolam does not contain propylene glycol, a solvent used to deliver medications such as lorazepam and pentobarbital that can cause severe metabolic acidosis [35]. Midazolam undergoes extensive hepatic metabolism via hydroxylation (CYP 3A4 and 3A5), to form active metabolite 1-hydroxymidazolam. This active metabolite is renally excreted, resulting in accumulation and prolonged effect in patients with renal dysfunction [36]. Other common side effects include respiratory depression, tachyphylaxis, and hypotension [37]. Although the 2012 Neurocritical Care Society Guidelines recommend an initial bolus dose of 0.2 mg/kg followed by a continuous infusion of 0.05–2 mg/kg/h, continuous infusion rates up to 2.9 mg/kg/h have been reported as safe and effective by a 2014 case series [34].

#### 3.2.2. Propofol

Propofol (Class IIb, level B) is a fast-acting anesthetic with a shorter half-life than midazolam. It also has activity at the GABA_A_ receptor, with the addition of NMDA inhibition [38]. Propofol utilization is largely limited by the increased incidence of side effects observed at the higher dosing needed for RSE compared with typical ICU sedation. These side effects include hypotension, hypertriglyceridemia, and propofol related infusion syndrome [39]. For RSE, propofol is administered as a loading dose of 1 to 2 mg/kg, followed by a continuous infusion of 30–200 mcg/kg/min. Additionally, propofol is formulated in a 10% fat emulsion containing 1.1 kcal/mL, so triglycerides should be monitored and dietary caloric intake must be adjusted based on infusion rates [2,40].

#### 3.2.3. Ketamine

Another attractive continuous anesthetic is ketamine due to its NMDA antagonism and its favorable side effect profile [41,42]. NMDA receptors are increasingly expressed during sustained seizure activity, offering a pathophysiological basis for the efficacy of ketamine in RSE and SRSE. In SRSE, ketamine has been studied retrospectively with a wide range of reported efficacy between 28–91% [41,43,44,45,46].

A recent, retrospective, single-center study evaluated ketamine use in 68 consecutive SRSE patients. All patients received midazolam infusions in addition to ketamine. Additionally, 11 patients had cerebral physiologic data monitoring. Within 24 h of ketamine initiation, 81% of patients had at least a 50% decrease in seizure burden. The average ketamine infusion rate was 2.2 mg/kg/h, with an average time to initiation of two days following admission. Importantly, ketamine was associated with stable mean arterial pressure and decreased vasopressor requirements over time, with no significant impact on conventional measures of brain physiology: intracranial pressure, cerebral perfusion pressure, and cerebral blood flow [41].

While there is limited evidence regarding optimal ketamine dosing for SRSE, loading doses of 0.5–5 mg/kg and continuous infusion rates of 1–10 mg/kg/h have been reported in the literature Although the optimal time to initiate ketamine also remains unknown, more recent literature seems to suggest better efficacy with earlier initiation [47]. Side effects of ketamine include both hyper- and hypotension, tachy- and bradycardia, cardiac arrhythmias, metabolic acidosis, hypersalivation, and an emergence phenomenon upon discontinuation. Ketamine should therefore be used with caution in patients with a history of severe cardiovascular disease. Although case reports and cases series previously reported an association between ketamine use and increased intracranial pressure, two large systematic reviews have since refuted the association. Of note, these reviews focused on bolus dosing in the operating room, rather than the high dose continuous infusions used for SRSE [48,49]. In an 11 patient sample with intracranial pressure monitoring, a single center study found no effect of high dosages of ketamine infusion on intracranial pressure [41].

#### 3.2.4. Barbiturate

Pentobarbital (Class IIb, level B) enhances chloride channel opening by binding to GABA_A_ receptors, thereby inhibiting cortical function, and inhibiting AMPA receptors [38]. It is administered as a loading dose of 5–15 mg/kg followed by a continuous infusion of 0.5–5 mg/kg/h pentobarbital use is largely limited by its high risk and occasionally fatal side effect profile, and therefore is typically reserved for SRSE resistant to other continuous anesthetics. Major reported side effects include hypotension, cardiac depression, respiratory depression, paralytic ileus, and metabolic acidosis secondary to propylene glycol [2,50].

One retrospective cohort study characterized 31 patients treated with continuous intravenous pentobarbital for SRSE, excluding post-anoxic SE [51]. The treatment successfully stopped SE in 90% of patients, nearly 40% of whom had suspected encephalitis. A maintenance dose of pentobarbital of 0.5–1.7 mg/kg/h was required to achieve burst suppression in 64% of patients, while 36% required doses of up to 2.0–3.7 mg/kg/h. Weaning resulted in withdrawal seizures in nearly half of patients, 80% of whom were adequately controlled with phenobarbital during weaning. Approximately 65% experienced side effects related to pentobarbital infusion, most commonly ventilator-associated pneumonia and hypotension in one third of patients. Only 10% of patients treated for SRSE had no or minimal disability one year after discharge [51].

In a smaller cohort of ten patients, five patients achieved seizure cessation. All patients had suspected viral encephalitis. Seven patients experienced hypotension, while five had pneumonia. Weaning was successful in five patients, and only one patient exhibited full recovery at discharge [52].

### 3.3. Non-Pharmacological Treatment of RSE and SRSE

In addition to pharmacological treatment of RSE and SRSE, multiple non-pharmacological options have also been investigated. These therapies include immunotherapy, neuromodulation, hypothermia, ketogenic diet, and finally surgical intervention in select cases.

Immunotherapy should be considered early in the treatment of new onset RSE, especially when suspecting autoimmune encephalitis [14,53,54]. Subacute onset of seizures with possible neuropsychiatric symptoms, as well as high seizure frequency, multifocal localization, and history of cancer or autoimmune disease, should raise suspicion for inflammatory or autoimmune etiology of RSE/SRSE [40]. Workup includes: MRI, lumbar puncture, and screening for neoplasm by PET scan, CT chest/abdomen/pelvis, pelvic and vaginal ultrasound, and abdominal MRI. Once infection is ruled out, clinicians should start high-dose corticosteroids, and consider early course of intravenous immunoglobulin or plasmapheresis. Certain autoimmune antibodies respond better with nonsteroidal immunomodulation medication [55].

Neuromodulation techniques were evaluated in a systematic review of case reports and case series with a high success rate for seizure control (more than 80%). Neuromodulation techniques include: electroconvulsive therapy, vagal nerve stimulation, and deep brain stimulation. These results should be interpreted cautiously due to publication bias risk [56].

Another attractive option is the use of ketogenic diet as ketone bodies have demonstrated anticonvulsant features possibly by increasing glutamine, and GABA synthesis [57]. The use of ketogenic diet has been used in patients with SLC2A1 (GLUT 1) mutations.

In a small retrospective case review involving 11 patients with SRSE, more than 90% of patients achieved resolution after ketogenic diet initiation [58]. In another small retrospective study in 16 patients who received ketogenic diet for SRSE, nine patients achieved complete resolution of seizure activity, while six patients achieved 50% reduction in seizure frequency [59]. In a multicenter study, ketogenic diet was started in patients with SRSE with ketosis state reached on average after two days, and successfully stopped SRSE in 73% of patients [60]. The ketogenic diet is contraindicated in patients with hepatic failure, acute pancreatitis, or metabolic acidosis [61,62]. It is also recommended not to combine the diet with propofol, since it increases the risk for fatal propofol related infusion syndrome [63].

The HYBERNATUS multicenter randomized controlled trial studied the efficacy of hypothermia (target temperature 32–34 °C for 24 h) as add-on therapy in critically ill, ventilated patients with convulsive SE hypothermia did not improve outcomes at three months [64]. Common adverse effects included deep vein thrombosis, coagulopathy, increase risk for infection, and hemodynamic alterations, bradycardia and hypotension [65].

Surgical intervention is an option in select cases to treat SRSE. Identifying the epileptogenic zone either by neuroimaging or EEG helps determine the type of surgical intervention needed, especially with partial epilepsy that originates from a lesion. A systematic review article noted that out of 36 cases identified, 33 received surgical intervention that led to complete control of SE [10,66].

## 4. Conclusions

RSE and SRSE are life-threatening conditions that require prompt diagnosis and treatment. Prompt treatment with ASM and anesthetics is critical. Non-pharmacological therapies are available and should be considered early in the course of treatment.

## Figures and Tables

**Table 1:** Commonly used ASMs for the treatment of refractory and super-refractory status epilepticus (RSE and SRSE).

ASM	Mechanism of Action	Loading Dose	Maintenance Dose	Metabolism	Adverse Effects	Comments
Clobazam	GABA_A_ agonist	variable	20 mg/d in 2 divided doses (range 10–60 mg/d)	Hepatic to active metabolite	Ataxia Somnolence/sedation Upper respiratory infections	Oral/enteral administration only
Fosphenytoin	Increases efflux/decreases influx of sodium across cell membrane	20 mg Phenytoin equivalents/kg IV (max 1500 mg)	4–6 mg Phenytoin equivalents /kg/d in divided doses	Rapid hydrolysis to phenytoin then hepatic	Arrhythmias Hypotension	Administer no faster than 150 mg PE/min Potent CYP inducer resulting in many drug-drug interactions Therapeutic drug monitoring required (total phenytoin level 10–20 mcg/mL or free phenytoin level 1–2 mcg/mL); total level unreliable in hypoalbuminemia and renal impairment Highly protein bound Transition to oral phenytoin when applicable
Lacosamide	Enhances slow inactivation of voltage gated sodium channels	200–400 mg IV	200–400 mg/d in 2 divided doses	Hepatic to inactive metabolites	Hypotension PR prolongation	Cardiac monitoring recommended with higher doses and in patients with history of cardiac disease
Levetiracetam	Unknown; may interact with N- type calcium channels, facilitates GABA inhibition, interacts with potassium rectifier current, and/or bind to synaptic vesicle proteins	60 mg/kg IV (max 4.5 g)	1000–3000 mg/d in 2 divided doses	Hydrolysis	Agitation/behavior disturbances Somnolence/sedation	Minimal drug interactions and adverse effects
Phenobarbital	Increases GABA activity by altering inhibitory synaptic transmission mediated by GABA_A_	20 mg/kg IV	1–3 mg/kg/d in divided doses	Hepatic to inactive metabolites	Hypotension Respiratory depression	Contains propylene glycol Therapeutic drug monitoring required (15–40 mcg/mL)
Perampanel	AMPA receptor antagonist	variable	Variable; 2–12 mg/d per package insert	Hepatic to inactive metabolites	Serious psychological and behavioral disturbances including SI (BW)	Major substrate o CYP 3A4 so higher doses may be required with inducers such as carbamazepine, phenytoin, or oxcarbazepine
Topiramate	Block voltage gated sodium channels, increase GABA activity, antagonize AMPA/kainite glutamate receptors, weak carbonic anhydrase inhibitor	Variable	300–1600 mg/d in divided doses	Not extensively metabolized	Metabolic acidosis Somnolence	Oral/enteral administration only
Valproic Acid	Increases availability of GABA or may increases the action of GABA; prolongs recovery phase of voltage-gated sodium channels	40 mg/kg IV (max 3000 mg)	10–60 mg/kg/d in divided doses	Hepatic to active metabolites	Hyperammonemia Pancreatitis Thrombocytopenia Transaminitis	Therapeutic drug monitoring required (50–100 mcg/mL) Concomitant use with carbapenem antibiotics should be avoided due to significant and prolonged drops in serum valproic acid level Highly protein bound

ASM: anti-seizure medication; BW: black box warning CYP: cytochrome P450; IV: intravenous; PE: phenytoin equivalents; SI: suicidal ideations.

**Table 2:** Continuous anesthetic infusions for the treatment of RSE and SRSE.

Anesthetic	Bolus Dose	Continuous Infusion Dose	Metabolism	Adverse Effects	Comments
Midazolam	0.2 mg/kg	0.05–2.9 mg/kg/h	Hepatic to active metabolite	Hypotension Respiratory depression	Tachyphylaxis with prolonged use Accumulation of active metabolite in renal impairment
Ketamine	0.5–5 mg/kg	1–10 mg/kg/h	Hepatic to active metabolite and others	Cardiac arrhythmias Emergence phenomenon Hyper/hypotension Metabolic acidosis	Cautious use in patients with history of severe cardiovascular disease
Propofol	1–2 mg/kg	30–200 mcg/kg/min	Hepatic to inactive metabolites	Hypotension Propofol related infusion syndrome (PRIS) Respiratory depression	Must adjust daily caloric intake (1.1 kcal/mL)
Pentobarbital	5–15 mg/kg	0.5–5 mg/kg/h	Hepatic to inactive metabolites	Cardiac depression Hypotension Metabolic acidosis Paralytic ileus Respiratory depression	Contains propylene glycol

## Data Availability

Not applicable.
